# A Nomogram Model for Predicting the Response to Transcatheter Arterial Embolization in Patients With Symptomatic Hepatic Hemangioma

**DOI:** 10.3389/fmolb.2021.722864

**Published:** 2021-11-26

**Authors:** Qinqin Liu, Nan You, Jiangqin Zhu, Jing Li, Ke Wu, Zheng Wang, Liang Wang, Yinan Zhu, Huiying Gu, Xuehui Peng, Lu Zheng

**Affiliations:** ^1^ Department of Hepatobiliary Surgery, the Second Affiliated Hospital of Army Medical University, Chongqing, China; ^2^ Department of Biliary-Pancreatic Surgery, Sun Yat-Sen Memorial Hospital, Sun Yat-Sen University, Guangzhou, China

**Keywords:** nomogram, therapeutic response, transcatheter arterial embolization, symptomatic hepatic hemangioma, efficacy

## Abstract

**Background:** Transcatheter arterial embolization (TAE) is regarded as an effective treatment for patients with symptomatic hepatic hemangioma. However, few studies have evaluated the efficacy of TAE alone for treating hepatic hemangioma. The aim of this study was to identify the factors that influence the response to TAE and formulate a quantitative nomogram to optimize the individualized management of hepatic hemangioma.

**Methods:** We retrospectively studied 276 patients treated with TAE for hepatic hemangioma at our center from January 2011 to December 2019. The full cohort was randomly divided into training and validation cohorts. After assessing the potential predictive factors for the efficacy of TAE in the training cohort, a nomogram model was established and evaluated by discrimination and calibration.

**Results:** During follow-up, the symptom relief rate was 100%. The tumor blood supply (*p* < 0.001), tumor number (*p* = 0.004), and tumor size (*p* = 0.006) were identified as significant predictors of the failure of tumor shrinkage in response to TAE. The nomogram model showed favorable discrimination and calibration, with a C-index of 0.775 (95% CI, 0.705–0.845) in the training cohort, which was further confirmed in the validation cohort (C-index 0.768; 95% CI, 0.680–0.856). The side effects of TAE were relatively minor and included mainly abdominal pain, nausea, vomiting, fever, and the presence of elevated hepatic transaminases.

**Conclusion:** TAE is a safe and effective treatment for symptomatic hepatic hemangioma. The established nomogram performed well for the estimation of the effect of TAE in patients with hepatic hemangioma and can facilitate the selection of patients who would benefit most from the treatment.

## Introduction

Hepatic hemangioma is the most common benign vascular lesion of the liver with an approximate incidence ranging from 0.4 to 20% (Choi and Nguyen, 2005; Fang et al., 2015). Hepatic hemangioma is congenital vascular malformations caused by vascular developmental abnormalities and abnormal differentiation, which expands during pregnancy and may be associated with the administration of estrogen (Chatzoulis et al., 2008; Duxbury and Garden, 2010; Ketchum et al., 2019). Most cases are found by chance with a high prevalence of accurate and sensitive imaging modalities. Small and asymptomatic lesions require no active treatment or monitoring (Herman et al., 2005; Schnelldorfer et al., 2010; Hasan et al., 2014). However, a small number of hepatic hemangiomas grow progressively and present as abdominal pain, distention, fatigue, anemia, obstructive jaundice, Kasabach–Merritt syndrome, and spontaneous rupture, which require clinical management (Donati et al., 2011; Toro et al., 2014; Liu et al., 2018).

The optimal selection of management strategies for expanding and symptomatic hepatic hemangioma remains controversial (Özden et al., 2017). Among them, transcatheter arterial embolization (TAE) is a promising, minimally invasive technique for the treatment of symptomatic hepatic hemangioma, associated with symptom relief and marked tumor shrinkage (Srivastava et al., 2001). A multicenter study involving 836 cases reported that transarterial chemoembolization was safe and effective for treating giant hepatic hemangioma, and the results showed a 100% symptom relief rate and clear tumor regression (Li et al., 2015). Several studies have investigated the effect of TAE on hepatic hemangioma, but most of them were limited by a small number of patients and short-term data (Akhlaghpoor et al., 2018; Torkian et al., 2021). Nevertheless, different treatment responses were not assessed in these previous studies and patients who would benefit from TAE were not well defined. In addition, one recent study attached the importance to preoperative TAE for treating ruptured hepatic hemangioma, which showed reduced blood loss in the subsequent surgical resection (Ramachandran et al., 2010). Despite the promising results in controlling hepatic hemangioma, some patients experienced failure to benefit from TAE. The present study aimed to investigate the potential factors correlating with the efficacy of TAE and to develop a nomogram for the management of individual hepatic hemangioma.

## Materials and Methods

### Patients

A cohort of 276 patients diagnosed with hepatic hemangioma, who underwent TAE only from January 2011 to December 2019 in the Second Affiliated Hospital of Army Medical University, were retrospectively collected. The inclusion criteria were as follows: 1) hepatic hemangioma confirmed by contrast-enhanced computed tomography (CT) or magnetic resonance imaging (MRI); 2) the presence of hepatic hemangioma–related symptoms and/or complications, such as abdominal complaints and intraabdominal hemorrhage; 3) Child–Pugh A liver function; 4) no other combined malignancies; 5) availability of complete clinicopathological and follow-up data; and 6) patients who did not receive other treatment options during the course of the disease. 276 eligible patients were ultimately enrolled and randomly divided into training (60%, n = 166) and validation (40%, n = 110) cohorts. The study was performed with approval from the Ethics Committee of the Second Affiliated Hospital of Army Medical University, and all patients signed written informed consent forms. All authors had access to the study data and reviewed and approved the final manuscript.

### TAE Procedure and Follow-Up

Under local anesthesia, all enrolled individuals underwent selective arteriography of their superior mesenteric, celiac, and common hepatic arteries to determine the location of the tumor and its blood supply. A mixture of pingyangmycin or bleomycin with lipiodol was slowly injected through a 5-Fr catheter into the tumor-feeding branches of the blood vessels. The amount of mixture injected depended on the size and number of the lesions. Embolization was conducted until complete tumor arterial flow stasis was observed on an angiogram.

The following variables were included for analysis: age, sex, oral contraceptives, hepatitis B surface antigen (HBsAg), comorbidities, tumor location, tumor distribution, tumor size, tumor number, tumor blood supply, white blood cell (WBC), platelet (PLT), hemoglobin (HGB), total bilirubin (TBil), alanine aminotransferase (ALT), aspartate transaminase (AST), drugs used for TAE, the number of TAEs, follow-up time, and postoperative complications. In addition, hemangioma-related symptom relief was assessed and recorded.

Every enrolled patient received the CT scan 1 month after the initiation of treatment and then every 3–6 months thereafter to evaluate the therapeutic response. The tumor response to TAE was based on the Response Evaluation Criteria in Solid Tumors (RECIST) (Duffaud and Therasse, 2000), and all patients were stratified into an effective group (complete response, CR, + partial response, PR) and an ineffective group (stable disease, SD, + progressive disease, PD). The TAE procedure was repeated according to the therapeutic efficacy and patient tolerance.

### Tumor Blood Supply

The CT value was measured at the largest cross-section of the tumor in the arterial enhancement phase before TAE, and the average CT value of all the tumors (CT1) was obtained. To better evaluate the blood supply of the lesions, the liver parenchyma CT value (CT2) in the arterial enhancement phase was measured. Subsequently, tumor blood supply was categorized as hypovascular or hypervascular based on the median CT1/CT2 value.

### Model Construction and Evaluation

The patients were randomly assigned into the training and validation cohorts with a split ratio of 3:2. Univariate and multivariate analyses were performed on the training cohort to determine the significant independent factors for which a predictive nomogram was indicated. The discrimination ability of the nomogram was determined using the concordance index (C-index). The calibration curve for predictive accuracy of the nomogram was used to analyze the consistency between the predicted and observed probability.

### Statistical Analysis

Statistical analyses were performed using SPSS version 22.0 (IBM SPSS, Inc., Chicago, IL) and R version 3.5.1 (http://www.r-proje ct.org). The continuous variables were summarized using the median with the interquartile range and compared using the *t*-test or Mann–Whitney–Wilcoxon test, while the categorical variables were expressed as frequency with proportion and compared using the chi-square test or Fisher’s exact test. Univariate and multivariate logistic regression analyses were employed to identify the significantly associated factors. Variables with *p* < 0.05 in the univariate analysis were entered into the multivariate analysis. A nomogram was constructed based on the independent predictors identified above using the rms R package. The discrimination and calibration of the nomogram were assessed using the C-index and the calibration curve. *p* values < 0.05 were considered statistically significant.

## Results

### Baseline Characteristics

In total, 276 patients who received TAE for hepatic hemangioma were enrolled in this study. Baseline characteristics of the training and validation cohorts are presented in Table 1. No statistically significant differences were observed between the two cohorts regarding tumor response to TAE, with response rates of 51.8 and 45.5% in the training and validation cohorts, respectively (*p* = 0.301). Tumor size, tumor number, and tumor blood supply differed significantly between the two groups in both the training and validation cohorts, and a larger number of patients were repeatedly treated with TAE in the ineffective group of the training cohort. The median follow-up duration was 3.4 (2.0–5.3) years for patients in the training cohort and 3.3 (1.4–5.3) years for patients in the validation cohort (*p* = 0.832).

**TABLE 1:** Demographic characteristics of patients with hepatic hemangioma in the training and validation cohorts.

Variables	Training cohort (n = 166)		*P*	Validation cohort (n = 110)		*P*
	Effective	Ineffective		Effective	Ineffective	
Age, years	46.0 (40.0–53.3)	46.0 (41.3–51.8)	0.911	47.0 (41.0–52.3)	45.0 (39.0–52.8)	0.465
Sex	—	—	0.490	—	—	0.123
Male	29 (33.7%)	23 (28.8%)	—	23 (46.0%)	19 (31.7%)	—
Female	57 (66.3%)	57 (71.3%)	—	27 (54.0%)	41 (68.3%)	—
Oral contraceptives	—	—	0.245	—	—	0.310
Yes	7 (8.1%)	11 (13.8%)	—	5 (10.0%)	10 (16.7%)	—
No	79 (91.9%)	69 (86.3%)	—	45 (90.0%)	50 (83.3%)	—
HBsAg	—	—	0.530	—	—	0.347
Positive	12 (14.0%)	14 (17.5%)	—	8 (16.0%)	6 (10.0%)	—
Negative	74 (86.0%)	66 (82.5%)	—	42 (84.0%)	54 (90.0%)	—
Comorbidities	—	—	0.484	—	—	0.515
Present	6 (7.0%)	8 (10.0%)	—	4 (8.0%)	2 (3.3%)	—
Absent	80 (93.0%)	72 (90.0%)	—	46 (92.0%)	58 (96.7%)	—
Tumor location	—	—	0.160	—	—	0.996
Left lobe	49 (57.0%)	40 (50.0%)	—	26 (52.0%)	31 (51.7%)	—
Right lobe	16 (18.6%)	10 (12.5%)	—	8 (16.0%)	10 (16.7%)	—
Bilobar	21 (24.4%)	30 (37.5%)	—	16 (32.0%)	19 (31.7%)	—
Tumor distribution	—	—	0.132	—	—	0.167
Subcapsular	23 (26.7%)	22 (27.5%)	—	20 (40.0%)	14 (23.3%)	—
Deep situated	56 (62.8%)	41 (51.3%)	—	24 (48.0%)	36 (60.0%)	—
Both	9 (10.5%)	17 (21.3%)	—	6 (12.0%)	10 (16.7%)	—
Tumor size, cm	6.1 (4.8–7.2)	6.7 (5.5–8.1)	0.020	6.0 (4.8–7.5)	6.9 (5.3–8.0)	0.023
Tumor number	—	—	<0.001	—	—	0.001
Solitary	56 (65.1%)	30 (37.5%)	—	32 (64.0%)	20 (33.3%)	—
Multiple	30 (34.9%)	50 (62.5%)	—	18 (36.0%)	40 (66.7%)	—
Tumor blood supply	—	—	<0.001	—	—	0.001
Hypovascular	26 (30.2%)	51 (63.8%)	—	19 (38.0%)	42 (70.0%)	—
Hypervascular	60 (69.8%)	29 (36.3%)	—	31 (62.0%)	18 (30.0%)	—
WBC, 10^9^/L	5.2 (4.5–5.9)	5.0 (4.3–6.0)	0.525	5.5 (4.5–6.3)	5.0 (4.4–6.1)	0.515
PLT, 10^9^/L	188.0 (138.8–230.0)	174.5 (146.3–213.3)	0.439	175.0 (141.0–208.8)	198.5 (162.0–221.3)	0.089
HGB, g/L	132.0 (121.8–142.0)	127.5 (118.0–140.0)	0.189	132.5 (119.8–143.0)	127.0 (114.3–138.0)	0.107
TBIL, umol/L	11.9 (9.1–15.6)	13.1 (9.3–17.3)	0.616	12.0 (9.2–15.7)	12.1 (9.9–17.6)	0.307
ALT, IU/L	19.0 (13.8–28.0)	16.0 (12.1–25.2)	0.121	19.0 (13.0–27.5)	16.0 (12.2–23.4)	0.174
AST, IU/L	18.0 (16.0–24.3)	18.1 (15.2–23.0)	0.634	19.5 (15.8–24.0)	18.0 (15.9–22.5)	0.644
Drug	—	—	0.617	—	—	0.152
Pinyangmycin	58 (67.4%)	51 (63.8%)	—	38 (76.0%)	38 (63.3%)	—
Bleomycin	28 (32.6%)	29 (36.3%)	—	12 (24.0%)	22 (36.7%)	—
Repeated TAE	—	—	0.030	—	—	1.000
0	85 (98.8%)	72 (90.0%)	—	49 (98.0%)	58 (96.7%)	—
1	1 (1.2%)	8 (10.0%)	—	1 (2.0%)	2 (3.3%)	—

### Complication and Symptomatic Improvement

The side effects of embolization were assessed in the present study, including abdominal pain in 167 cases (60.5%), nausea or vomiting in 124 cases (44.9%), fever in 73 cases (26.4%), and elevation of hepatic transaminases in 31 cases (11.2%). These symptoms were limited to 3–4 days and required no treatment or were easily controlled with symptomatic treatment. No serious complications were observed. All patients showed reduced symptoms from the intervention of TAE.

### Univariate and Multivariate Analyses of the Predictive Factors Associated With Tumor Response to TAE in Patients With Hepatic Hemangioma

The univariate and multivariate analyses of the training cohort were employed to investigate the predictors of the response to TAE in patients with hepatic hemangioma (Table 2). By assessing multiple potential predictive factors, the variables of tumor size, tumor number, tumor blood supply, and repeated TAE were determined to be related to the efficacy of TAE. Furthermore, the results of the multivariate analysis showed that tumor blood supply (HR = 5.150; 95% CI = 2.468–10.743; *p* < 0.001), tumor number (HR = 2.825; 95% CI = 1.402–5.695; *p* = 0.004), and tumor size (HR = 1.293; 95% CI = 1.075–1.554; *p* = 0.006) were significant predictors of the failure of tumor shrinkage in response to TAE.

**TABLE 2:** Univariate and multivariate analyses of predictive factors associated with tumor response to TAE in the training cohort.

Variables	Univariate analysis	*p*	Multivariate analysis	*p*
	Hazard ratio (95%CI)		Hazard ratio (95%CI)	
Age, years	0.997 (0.964–1.031)	0.853	—	—
Sex	—	—	—	—
Male	Reference	—	—	—
Female	1.261 (0.652–2.437)	0.491	—	—
Oral contraceptives		—	—	—
No	Reference	—	—	—
Yes	1.799 (0.661–4.896)	0.250	—	—
HBsAg	—	—	—	—
Negative	Reference	—	—	—
Positive	1.308 (0.565–3.028)	0.531	—	—
Comorbidities	—	—	—	—
Absent	Reference	—	—	—
Present	1.481 (0.491–4.474)	0.486		—
Tumor location	—	—	—	—
Left lobe	Reference	—	—	—
Right lobe	0.766 (0.313–1.871)	0.558	—	—
Bilobar	1.750 (0.872–3.513)	0.115	—	—
Tumor distribution		—	—	—
Subcapsular	Reference	—	—	—
Deep situated	0.794 (0.390–1.617)	0.525	—	—
Both	1.975 (0.728–5.353)	0.181	—	—
Tumor size, cm	1,229 (1.051–1.437)	0.010	1.293 (1.075–1.554)	0.006
Tumor number	—	—	—	—
Solitary	Reference	—	Reference	—
Multiple	3.111 (1.651–5.863)	<0.001	2.825 (1.402–5.695)	0.004
Tumor blood supply		—	—	—
Hypervascular	Reference	—	Reference	—
Hypovascular	4.058 (2.123–7.756)	<0.001	5.150 (2.468–10.743)	<0.001
WBC, 10^9^/L	0.869 (0.707–1.068)	0.182	—	—
PLT, 10^9^/L	0.998 (0.993–1.003)	0.386	—	—
HGB,g/L	0.990 (0.971–1.009)	0.287	—	—
TBIL,umol/L	1.024 (0.970–1.078)	0.392	—	—
ALT,IU/L	0.992 (0.976–1.008)	0.310	—	—
AST,IU/L	0.995 (0.966–1.024)	0.714	—	—
Drug	—	—	—	—
Pinyangmycin	Reference	—	—	—
Bleomycin	1.178 (0.620–2.237)	0.617	—	—
Repeated TAE	—	—	—	—
0	Reference	—	Reference	—
1	9.444 (1.154–77.312)	0.036	7.670 (0.756–77.850)	0.085

### Nomogram Construction and Validation

**FIGURE 1:**
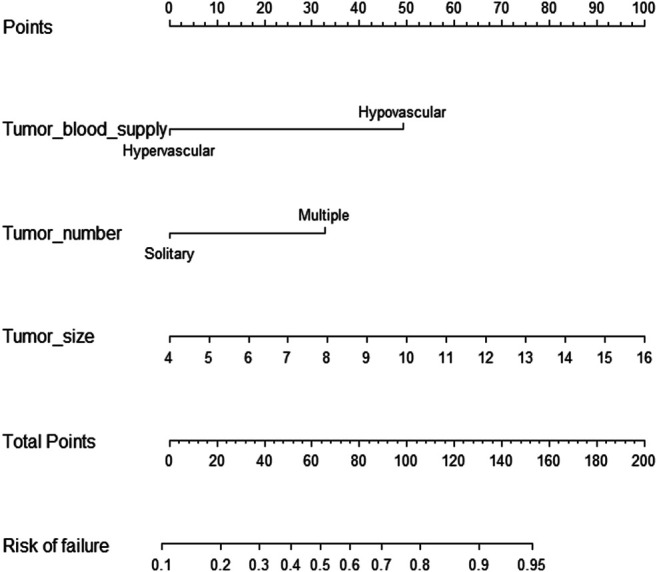
Nomogram for predicting the probability of tumor response to TAE in a patient with hepatic hemangioma.

**FIGURE 2:**
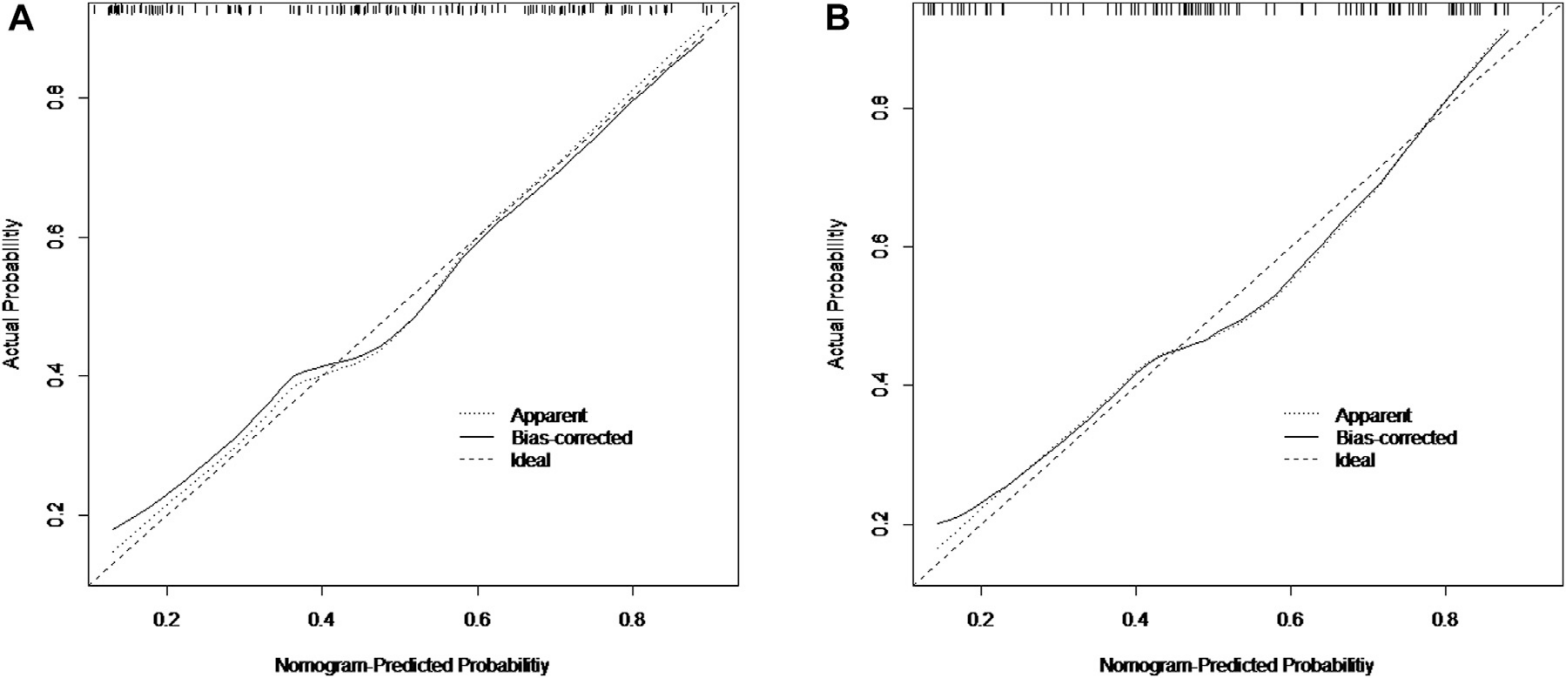
Calibration curves of the nomogram for the training cohort **(A)** and the validation cohort **(B)**.

The nomogram incorporating the independent variables, namely, tumor size, tumor number, and tumor blood supply, identified above, was constructed to estimate patients’ personalized therapeutic response to TAE (Figure 1). The nomogram yielded a C-index of 0.775 (95% CI, 0.705–0.845) for the training cohort and a C-index of 0.768 (95% CI, 0.680–0.856) for the validation cohort. Based on the projecting total score onto the lower total point scale by adding the scores of each variable, the probability of tumor response to TAE in a patient with hepatic hemangioma can be estimated. The calibration curve indicated the optimal agreement between nomogram-predicted probability and the result of observation in both the training and validation cohorts (Figure 2).

## Discussion

Symptomatic hepatic hemangioma is a common hepatic neoplasm that requires effective intervention. Among various treatment options for patients with hepatic hemangioma, TAE is accepted as a less invasive method associated with fewer complications and quicker recovery (Szejnfeld et al., 2015). However, not all patients respond well to TAE, evidenced by the heterogeneous tumor response. In this study, we analyzed the clinical data of 276 patients who received TAE for symptomatic hepatic hemangioma, and an efficacy estimation nomogram was created. To the best of our knowledge, the study is the first exploration of the potential therapeutic effect on TAE in patients with hepatic hemangioma.

Surgical resection is technically difficult and associated with severe complications and a long recovery time (Yoon et al., 2003; Miura et al., 2014). Although microwave ablation and radiofrequency ablation are thought to be less risky treatments, the long ablation time and dangerous hemolysis may reduce the intervention benefits for hepatic hemangioma (Gao et al., 2013; Ji et al., 2016). In comparison, TAE is more applicable, and clinical remission and tumor size reduction can be observed. In addition, TAE can be used to downsize a giant hepatic hemangioma, thus facilitating the subsequent resection or ablation, thereby minimizing possible complications (Firouznia et al., 2014).

Our analysis demonstrated that there was improvement in the symptoms of all patients with symptomatic hepatic hemangioma treated with TAE, which is consistent with the findings of previous studies (Deutsch et al., 2001; Srivastava et al., 2001). Given that the shrinkage of hepatic hemangioma in response to TAE differed among patients, the potential predictive variables were further analyzed. This involved determining the significant pretreatment parameters that could be used for tumor response prediction. The nomogram was developed further to predict each patient’s likelihood of response to TAE based on the factors, which showed good predictive performance with a C-index of 0.775 (95% CI, 0.705–0.845) for the training cohort, which was validated by the internal validation cohort with a C-index of 0.768 (95% CI, 0.680–0.856). Thus, optimal candidates for TAE could be identified based on the individual probability of response to TAE. For lesions refractory to TAE, other suitable alternatives such as surgical resection should be considered.

In our nomogram model, tumor blood supply is identified as the greatest contributor to tumor response from TAE. The hypervascular lesion is significantly associated with increased efficacy of TAE. Previous studies indicated that hepatic hemangioma consists of blood-filled sinuses nourished by hepatic arteries (Giavroglou et al., 2003; Li et al., 2003). Superselective embolization of the multiple feeding vessels of the hepatic hemangioma allowed pinyangmycin or bleomycin to damage tumor-associated endothelial cells continuously and accelerate the progression of the occlusion and fibrosis of blood sinuses, thereby causing tumor regression (Szejnfeld et al., 2015). Thus, an improved accumulation of lipiodol is more likely to be achieved in a hypervascular lesion, resulting in increased probability of a positive therapeutic effect. Meanwhile, it is clear that hypovascular lesions are less sensitive to TAE because an insufficient quantity of embolic agents is deposited in the tumor.

Besides, tumor size and tumor number were associated with an increased risk of TAE treatment failure. Generally, TAE provides a hypoxic and anoxic environment to shrink the lesions by blocking the arterial blood supply (Xue et al., 2017). However, multifocal or giant hepatic hemangioma may recruit new blood vessels by angiogenesis and vasculogenesis, which reduces the long-term therapeutic efficacy in spite of repeated TAE. These cases with multiple feeding vessels, in which the effective dose of lipiodol is compromised by the tumor burden, are possible explanations for treatment failure. Although neovascularization may affect the tumor response, tumor shrinkage is progressive over time, and a satisfactory TAE response can be achieved in a single small lesion (Bozkaya et al., 2014). Consequently, other effective interventions or combined therapies are encouraged for patients with multinodular and large hepatic hemangioma.

The commonly reported side effects associated with TAE are abdominal pain, fever, nausea, and vomiting, which are caused by thrombosis and necrosis (Giavroglou et al., 2003). These complications were transient, most of which were resolved easily with conservative therapy (Li et al., 2015). Serious side effects rarely occur, such as liver failure, hepatic abscess formation, and cholecystitis. Given the benign nature of hemangioma, TAE is the preferred treatment, due to the good efficacy and its association with minor side effects when compared with the surgical approach (Deutsch et al., 2001). In agreement with our initial favorable experience with TAE, no major TAE-related complications occurred, and only mild and transient symptoms were noted.

Although the nomogram showed favorable predictive accuracy for the probability of poor tumor response to TAE in patients with hepatic hemangioma, there are still some limitations that should be acknowledged. First, this is a retrospective study from a single institution, which may introduce some potential bias. Second, the small sample size in the established nomogram may affect the accurate assessment of treatment failure. Third, there was no external validation, and further evaluation is required before applying the model in clinical practice. Therefore, a further prospective study with a larger sample size is warranted to confirm the validity of the model.

In conclusion, TAE is a safe and effective treatment option with satisfactory symptom control and marked tumor shrinkage in appropriately selected patients. A novel nomogram integrating independent predictive variables was developed to predict the efficacy of TAE in patients with hepatic hemangioma, which demonstrated good accuracy and calibration and could assist clinicians to develop an individualized treatment strategy.

## Data Availability

The original contributions presented in the study are included in the article, further inquiries can be directed to the corresponding author.

## Abbreviations

HBsAg, hepatitis B surface antigen; WBC, white blood cell; PLT, platelet; HGB, hemoglobin; TBil, total bilirubin; ALT, alanine aminotransferase; AST, aspartate transaminase; TAE, transcatheter arterial embolization.
